# Sources of get information and related factors during pregnancy among Afghan migrant women in Iran

**DOI:** 10.1002/nop2.707

**Published:** 2020-11-24

**Authors:** Mahnaz Sharifi, Leila Amiri‐Farahani, Nourossadat Kariman, Syedeh Batool Hasanpoor‐Azghady, Mina Amiri‐Farahani

**Affiliations:** ^1^ Department of Reproductive Health and Midwifery School of Nursing and Midwifery Iran University of Medical Sciences Tehran Iran; ^2^ Department of Reproductive Health and Midwifery Nursing Care Research Center School of Nursing and Midwifery Iran University of Medical Sciences Tehran Iran; ^3^ Midwifery and Reproductive Health Research Center School of Nursing and Midwifery Shahid Beheshti University of Medical Sciences Tehran Iran; ^4^ Shahid Chamran University of Ahvaz Ahvaz Iran

**Keywords:** information seeking, migrant, pregnant women, source of information

## Abstract

**Aim:**

The present study aims to investigate the sources of information and its related factors among pregnant Afghan migrant women who reside in southeast Tehran Province, Iran.

**Design:**

Cross‐sectional study.

**Methods:**

A total of 280 pregnant Afghan women who received care at the prenatal clinics of selected healthcare centres in southeast Tehran Province (Iran) in 2018 enrolled in this study. Data were collected by continuous sampling by a questionnaire that asked about demographic, obstetric and sources of information used during pregnancy.

**Results:**

The most important sources of information accessed by pregnant Afghan women were healthcare providers (65.1%), family and friends (47.55%), the Internet (32.1%) and media (18.9%). There was statistically a significant relationship between sources of information and education level, number of children, length of residence in Iran, place of birth and insurance status.

**What does this paper contribute to the global clinical community?**

- The results of this study identify sources of information and related factors in pregnant Afghan migrant women that might affect their decision‐making process when receiving healthcare services.
- The practical and significant results in this study can help improve the current situation for Afghan women. Policy makers can use this information to help the Afghan women make informed pregnancy‐related decisions based on information from appropriate sources.

## 1 INTRODUCTION

Although pregnancy is a natural occurrence for women, there is an obvious need for information during this time (Almalik & Mosleh, 2017; Clarke et al., 2016). Pregnancy is a critical period that can be used to promote health behaviour among women, especially those who reside in developing countries (Anya et al., 2008).

During pregnancy, women face many physical and mental changes, and they undergo many paraclinical tests. These women may have numerous questions; thus, they collect information from various sources in order to provide answers to their questions (Clarke et al., 2016). Access to this information is necessary to relieve their pregnancy‐related stress and concerns (Deave et al., 2008).

Searching and receiving information during pregnancy and the right to have relevant education are beneficial for people of all societies. In order to meet these rights, people's needs should be met, and anything that limits a woman's access to information should be questioned (Sanders & Crozier, 2018).

According to the National Institute of Health and Care Excellence policies, women should have access to information before, during and after pregnancy, in addition to information about newborn care and better decision‐making during pregnancy (Hildingsson et al., 2014). Women who have more information can play an effective role in the early diagnosis of disorders (Carolan, 2007). Information gathering about risk factors, especially for women who experience high‐risk pregnancies, improves foetal health, particularly in regions where there is inadequate access to prenatal care (Clarke et al., 2016). Mothers who have adequate awareness about pregnancy, childbirth and newborn care have higher self‐confidence and play more active roles during their pregnancy, labour and self‐care (Shieh et al., 2009). (Shieh et al., 2010).

Several studies have been conducted to understand immigrant mothers' health information needs, their searching behaviour and health information sources during pregnancy. Cultural needs and expectations are often unknown or not met in the context of the healthcare systems in the majority of host countries and may negatively impact the experiences of immigrant women and cause immigrant women to stop searching for information and turn to other, unreliable sources (Khanlou et al., 2017).

Bhagat and colleagues found that South Asian immigrant women were often uncomfortable expressing their needs or identifying gaps in the health care system (Bhagat et al., 2002). The authors of a large‐scale focus group study among low‐income mothers in the United States reported that participants in their study preferred face‐to‐face contact with healthcare providers or with other mothers when seeking health‐related information, advice and support. It would be more difficult for mothers to obtain information in countries where their native language is not spoken (Gazmararian et al., 2014). In another U.S. study, Lee reported that Korean immigrant mothers who resided in the United States most frequently used the Internet for information, followed by healthcare providers, books, newspapers and/or magazines, and TV programs. Friends, church members and family members were also key sources for Korean Americans who searched for health information (Lee, 2018).

Afghans are the largest migrant group in Iran; they have resided in Iran for more than three decades. There is a high rate of prenatal disorders attributed to the Afghan women's lack of information and awareness about pregnancy (Shafiei et al., 2012). Most often, migrant women are excluded from studies due to their inability to complete questionnaires or lack of familiarity with the host country's language. Hence, there is a decreased amount of information about the health‐related experiences of migrant women (Who, 2018). Searching for health information is highly important for immigrant mothers. Immigrants are more likely to actively seek a wide range of information in order to adjust to their new environment. Therefore, it is necessary to investigate the pregnancy health‐related information sources that are available for Afghan women in Iran in order to promote and improve their health and increase the quality of information sources available to them. The current study was designed because there is a lack of research in Iran about the sources of information needed during pregnancy and its related factors for Afghan women.

## 2 METHODS

This was a cross‐sectional study conducted with Afghan women who referred to selected perinatal clinics in southeast Tehran Province, Iran from May until July 2018. The sample size was determined to be 280 women, with an accuracy of d = 0.2 at a significance level of 95%. This study was performed according to the Strengthening the Reporting of Observational Studies in Epidemiology (STROBE) statement in File S1.

We performed continuous sampling during the study period, 5 days per week, in four healthcare centres in the southeast of Tehran Province. A total of 100 women from Varamin, 80 women from Pishva, 60 women from Pakdasht and 40 women from Qarchak cities were enrolled in this study. Inclusion criteria were as follows: current pregnancy, Afghan nationality and the presence of a health record at one of the four health clinics.

The data collection tool consisted of three parts: demographic data (age, education level, length of time at current residence, place of birth, place of residence and insurance), obstetric data (number of children, gestational age, routine prenatal care and previous delivery) and a question that asked about sources of information during pregnancy. This question consisted of four parts: Family, friends and relatives; the Internet (websites, Facebook, Instagram and Telegram); media (radio, TV, newspapers, magazines and books); and healthcare providers (midwives, general practitioners, gynaecologists and nurses). Women could choose more than one source of information. The answers to the questions were reported as percentages. The questionnaire was designed after a literature review, evaluation of the various tools that address sources of information during pregnancy, and according to viewpoints of some of the pregnancy health specialists. Both face and content validities were applied to validate the tool used to assess the source of information. Face validity was evaluated by face‐to‐face interviews with 30 Afghan women in order to determine the difficulty and ambiguity of the tool. Content validity was evaluated by corrective feedback from nine faculty members of the Department of Midwifery of Iran University of Medical Sciences, Tehran Iran and three midwifery specialists from Afghanistan.

### 2.1 Ethical consideration

The present research was approved by the Research Deputy of Iran University of Medical Sciences, Tehran, Iran after approval from the Ethics Committee of Iran University of Medical Sciences (ethical code: IR.IUMS.REC1396, 9513593001). The participants provided written informed consent for study participation, and the respondents were completely informed of the study purpose and procedures. All participants were assured of the confidentiality of their information.

### 2.2 Statistical analysis

IBM SPSS Statistics for Windows, version 21.0 (IBM Corp.), was used for data analysis. Demographic and obstetric characteristics were assessed with descriptive that included frequency distribution, central tendency and dispersion indicators such as mean and standard deviation. The chi‐square test was used to determine the relationship between the sources of information during pregnancy and demographic and obstetric characteristics. *p*‐values <.05 were considered to be statistically significant.

## 3 RESULTS

The mean and standard deviation of the participants' ages was 29.6 ± 6 years. There were 3.6% of the participants who had an academic education, and 26.8% had an elementary education. A total of 77.5% of the participants lived in a city, and 22.5% resided in a village. There were 60.7% of the participants were born in Afghanistan, and 39.3% were born in Iran. The average period of residing in Iran was 17.83 years. Only 12.1% had health insurance coverage. The average gestational age was 25 weeks. Natural vaginal delivery was reported by 77.8% of multipara women, and 22.2% had a caesarean section. A total of 57.7% of the participants had regular visits to healthcare providers to receive routine pregnancy care. The most common source of information was healthcare providers for 65.1% of participants, and the least common source of information was the media (18.9%). Table 1 lists the frequency of using these sources. Table 2 provides additional information about the relationship between demographic and obstetric characteristics, and information sources.

**TABLE 1:** Frequency of the source of information accessed by pregnant Afghan women

Source of information	*N* (%)
Media	53 (18.9)
Family and friends	133 (47.5)
Healthcare provider	182 (65.1)
Internet	90 (32.1)

**TABLE 2:** Relationship between demographic and obstetric characteristics and use of information sources by the pregnant Afghan women

Variables	Healthcare provider Yes, *N* (%)	Internet Yes, *N* (%)	Media Yes, *N* (%)	Family and friends Yes, *N* (%)
Education level				
Illiterate	51 (32.3)	4 (4.3)	8 (14.8)	42 (31.6)
Elementary	45 (28.5)	13 (14.1)	12 (22.2)	41 (30.8)
Junior high school	33 (20.9)	34 (37)	17 (31.5)	36 (27.1)
Senior high school	29 (18.4)	41 (44.6)	75 (84.5)	14 (10.5)
*p*‐value	.37	*<.001	*.005	*.01
Number of children				
0	51 (32.2)	49 (53.2)	27 (50)	46 (46.9)
1	71 (44.9)	35 (38.04)	22 (40.7)	66 (52.4)
2	25 (15.8)	6 (6.5)	3 (5.5)	16 (37.5)
3	10 (6.3)	2 (2.1)	2 (3.7)	5 (47.5)
*p*‐value	.30	*<.001	*.03	.14
Time of residence, year				
<5	29 (51.8)	8 (14.3)	6 (10.7)	29 (51.8)
5–10	16 (80)	3 (15)	1 (5)	12 (60)
11–15	8 (50)	5 (31.3)	4 (24)	10 (62.5)
16–20	40 (51.3)	24 (30.8)	15 (19.2)	41 (52.6)
>20	65 (59.1)	52 (47/3)	28 (25.5)	41 (37.3)
*p*‐value	.16	*<.001	.08	.14
Age, year				
<19	14 (50)	11 (39.3)	5 (17.9)	18 (64.3)
20–25	40 (46)	25 (40.2)	20 (23)	47 (54)
26–30	61 (66.3)	31 (33.7)	19 (20.7)	40 (43.5)
31–35	29 (63)	10 (21.7)	6 (13)	17 (37)
>35	14 (51.9)	5 (18.5)	4 (14.8)	11 (40.7)
*p*‐value	.06	.1	.66	.10
Gestational age, wk				
<15	17 (42.5)	10 (25)	8 (20)	18 (45)
15–28	70 (56.9)	46 (37.4)	26 (21.1)	53 (43.1)
>28	71 (60.7)	36 (30/8)	20 (17.1)	62 (53)
*p*‐value	.13	.28	.72	.29
Previous delivery				
Caesarean	27 (65.9)	12 (29.3)	7 (17.1)	14 (34.1)
Vaginal	82 (56.9)	31 (21.5)	21 (14.6)	74 (51.4)
None	49 (51.6)	49 (51.6)	26 (27.4)	45 (74.4)
*p*‐value	.30	*<.001	*.04	.14
Routine care				
Yes	129 (60.8)	76 (35.7)	45 (21.2)	94 (44.3)
No	29 (42.6)	16 (23.5)	9 (13.2)	39 (57.4)
*p*‐value	*.008	.06	.14	.06
Insurance				
Yes	139 (56.5)	74 (30.1)	43 (17.5)	133 (47.5)
No	19 (55.9	18 (52.9	11 (32.4)	12 (48.8)
*p*‐value	.94	*.008	*.03	.24
Place of residence				
Urban	124 (78.5)	73 (79.3)	42 (77.8)	98 (73.7)
Rural	34 (21.5	19 (20.7)	12 (22.2)	35 (26.3)
*p*‐value	.65	.60	.95	.14
Place of birth				
Iran	54 (44.3)	62 (67.4)	30 (55.6)	46 (34.6)
AFG	68 (55.7)	30 (32.6)	24 (44.4)	87 (65.4)
*p*‐value	.13	*.001	*.006	.65

## 4 DISCUSSION

According to the results of the present research, the pregnant Afghan women used the following sources for information: healthcare providers (65.1%), family and friends (47.5%), the Internet (32.1%) and media (18.9%). According to the results, the most common sources of information among the pregnant Afghan women were healthcare providers, family and friends. Like other women, Afghan women feel more comfortable with their family members because family members can better understand their condition and give suggestions that are closer to their culture (Adelkhah & Olszewska, 2007). Gazmararian et al. have conducted a study to identify the educational needs and resources used by first‐time mothers. The mothers and healthcare providers reported that the role of families, along with providing support during pregnancy was undeniable. In some cases, the advice given by family members, especially grandmothers, may be outdated and unprofessional; however, their advice closely approximates the pregnant women's living conditions and is more applicable in their lives (Gazmararian et al., 2014).

Grimes et al. conducted a study in Australia where women received a questionnaire that included different sources of information. The participants were asked to select the sources of information that they considered to be the most useful. According to these researchers, the sources of information used by women were midwives (70.3%), healthcentre booklets (61.1%), family and friends (52%), the Internet (44%), antenatal classes (42.6%), books (42.6%), written information provided by midwives (38.9%) and gynaecologists (12.6%). According to the participants' viewpoints, the most useful sources for them included books, midwives and the Internet, respectively. Non‐English speaking migrant women reported that midwives were the best sources of information (Grimes et al., 2014). In both the present study and the study by Grimes et al., the healthcare provider was the most common source of information used by pregnant women. In both studies, about 50% of the participants reported that family and friends were sources of information. This finding was similar in both studies. However, these studies were not consistent for books where Grimes et al. reported that 42.6% of women used books as sources of information compared with 18.9% of women who used media as an information source in the present study. Mostly, this was attributed to differences in the participants' education levels. In the Grimes et al. study, 74.3% of women had an academic education and there were no illiterate participants (Grimes et al., 2014). However, in the present research, only 3.6% of the participants had an academic education and 30% of the participants were illiterate. As a result, Afghan women tended to use written sources of information such as books less frequently than the participants in the Grimes et al. study. Hence, it would be better for Afghan immigrant women to get information from midwives who are healthcare centre employees because direct contact with an expert could give them an increased chance to find answers for their questions and fulfil their information needs (Guendelman et al., 2017).

In England, the most common sources of information used by migrant women were healthcare providers (88%), family and friends (72%) and the Internet (28%) (Soltani & Dickinson, 2005). In terms of the priority of the used sources of information, the results of the study performed by Soltani and Dickinson supported the findings of the present study. However, the results were inconsistent in terms of frequency. On the other hand, the higher rate of healthcare providers as sources of information in the present study was due to Afghan women's easy access to health centres and the free services provided for migrants in these centres.

There was a significant relationship between those who obtained information from their families and friends and those who did not, in terms of the women's education levels. Singh et al. reported that there was no relationship between women's demographic characteristics and the source of information. Only young women reported that their mothers were the most helpful source of pregnancy‐related information (Singh et al., 2002). In this regard, the results of the current study were not consistent with the findings by Singh et al. In the present study, about 47.3% of women reported that they received the necessary information from their families, friends and relatives. It is common in traditional communities to use the information of other women who have had previous pregnancy experiences. In Afghan communities, women are willing to use this source of information regardless of their age. In other words, there is no difference between women in terms of age and using this source of information. Singh et al. reported that migrants and minority groups were mainly willing to obtain information by phone consultations and social workers. We did not investigate this item because the use of a phone and referral to social workers for getting pregnancy information was not common.

A significant relationship existed between women who used the internet as a source of information and those who did not in terms of the number of children, their education level, birthplace, the period of time residing in Iran, type of previous delivery and insurance status. Grimes et al. reported that using the Internet as a source of information was more common among educated than uneducated women. There was no relationship between the use of the Internet and women's age according to Grimes et al. In terms of the relationship between age and source of information, our study supported the findings reported by Grimes et al. However, the two studies were not consistent in terms of the relationship between birthplace and the use of the Internet as a source of information. Grimes et al. reported that most women who were born outside of Australia and did not have any English background were willing to obtain information from the Internet (Grimes et al., 2014). In a study performed by Risica, there was no significant difference between primiparous and multiparous women in terms of Internet use (Risica & Phipps, 2006). However, in the present research, most women who were pregnant for the first time were willing to obtain information from the Internet. In this regard, the two studies were not consistent.

In the present study, we observed a significant relationship between women used the media as an information source and those who did not in terms of the number of children, their education level, birthplace, type of previous delivery and receiving routine pregnancy care. According to Grimes et al., the use of books as a source of information was more common among English speaking women born in Australia than non‐English speaking women (Grimes et al., 2014). In the present study, the use of books, newspapers, radio and TV as a source of information was more common among Afghan women born in Iran than those who were born in Afghanistan. Risica reported that no relationship existed between the type of previous delivery and the use of the Internet (Risica & Phipps, 2006). This finding was not consistent with the results of the present study.

In terms of receiving routine pregnancy care, we noted a statistically significant relationship between women who used health care providers as sources of information and those who did not. This relationship could be justified regarding the study performed by Grimes et al. These researchers stated that referrals to health centres increased access to healthcare providers and women usually used the most available sources to obtain information (Grimes et al., 2014).

### 4.1 Limitations

In the present study, there was a concern about the participants not reporting their actual sources of information. Therefore, in order to increase the accuracy of the answers, the researcher took several actions. First, the questionnaires were anonymous. Illiterate women were given blank questionnaires, and the researcher read the questions and answers out loud, and the answers were marked. Finally, the interview was performed in a quiet room.

The present research evaluated only the women's sources of information, and we did not investigate the way of seeking information. Therefore, we suggest that a study should be conducted to investigate the information‐seeking behaviours among pregnant Afghan women. The obstacles to access the sources of pregnancy information were not investigated in the present research. In addition, a study should be conducted to evaluate the obstacles faced by pregnant Afghan women when trying to access sources of information.

## 5 CONCLUSION

The findings of the current study confirmed that women actively search for health‐related information as part of their roles as mothers. When choosing the source of information, more than half are willing to refer to healthcare to get information, followed by family, friends and relatives. Furthermore, demographic (education level, the period of residing in Iran and insurance status) and obstetric characteristics (the number of children) affect the choices of information sources. The results of this study can help nurses and midwives to prioritize the type of information source with the demographic and obstetric characteristics of pregnant women when providing them with the necessary pregnancy‐related information.

## CONFLICT OF INTEREST

There is no conflict of interest.

## AUTHOR CONTRIBUTIONS

M.S, N.K and L.A.F: Study design. M.S and S.B.H: Data analysis and interpretation. M.S, M.A.F and L.A.F: Writing the manuscript and manuscript revision.

## RELEVANCE FOR THE CLINICAL PRACTICE

The results of this study can help nurses and midwives to prioritize the type of information source with the demographic and obstetric characteristics of pregnant women when providing them with the necessary pregnancy‐related information.

## Supporting information

File S1

## Data Availability

Data are available for use by contacting Dr. Amiri‐Farahani, l.amirifarahani@gmail.com.
